# Supplementary material for: Transient metal-centered states mediate isomerization of a photochromic ruthenium-sulfoxide complex
Source: Nat Commun. 2018 May 18;9:1989. doi: 10.1038/s41467-018-04351-0 (PMC5959936; doi:10.1038/s41467-018-04351-0)
Supplement: Supplementary file 1 — Supplementary Information [file 41467_2018_4351_MOESM1_ESM.pdf]

Supplementary Information

Transient Metal-Centered States Mediate Isomerization of a Photochromic Ruthenium-Sulfoxide Complex

Cordones et al.

Supplementary Figure 1. TD-DFT simulated spectra at the Ru L_3-edge for all ground states and possible intermediate excited states, including the primary orbitals involved for the most intense transitions. (a) S-bonded ground state ^1G_S. (b) S-bonded ^{3}MLCT state $^3MLCT_S$. (c) S-bonded ^{3}MC state ^3MC_S. (d) O-bonded ^{3}MC state ^3MC_O. (e) O-bonded ground state ^1G_O.

a

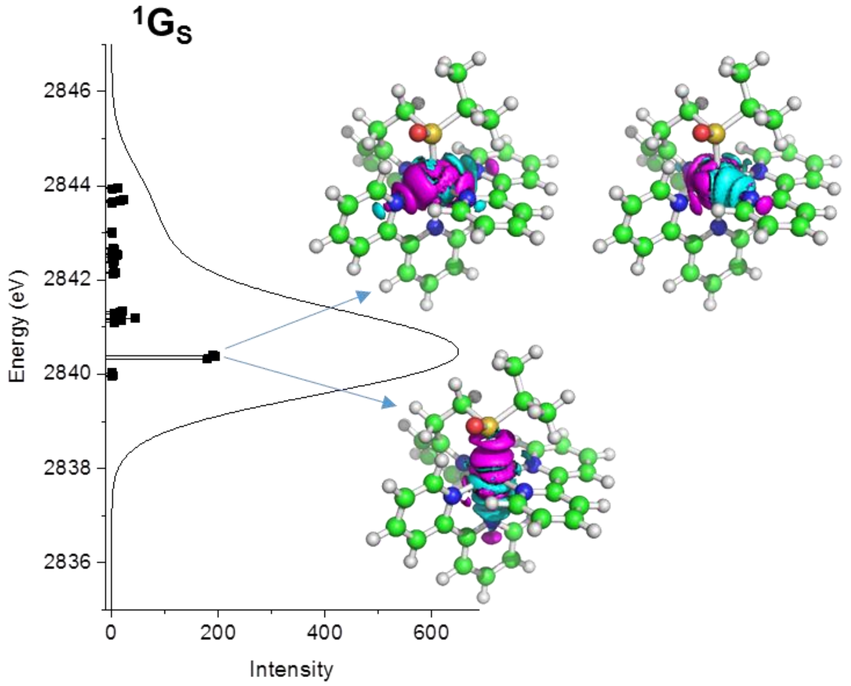

b

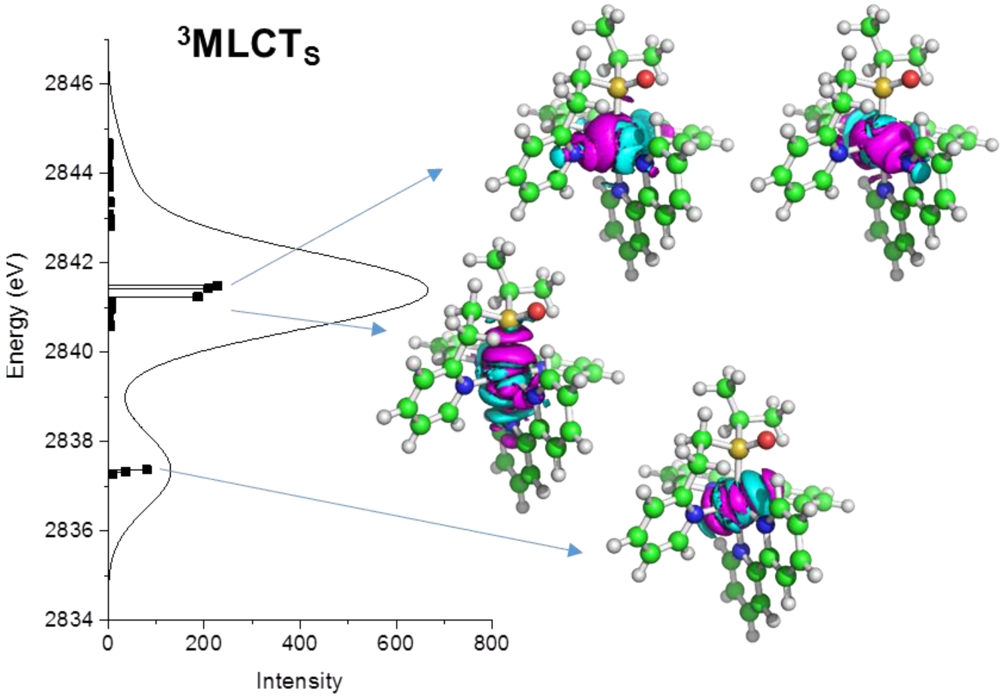

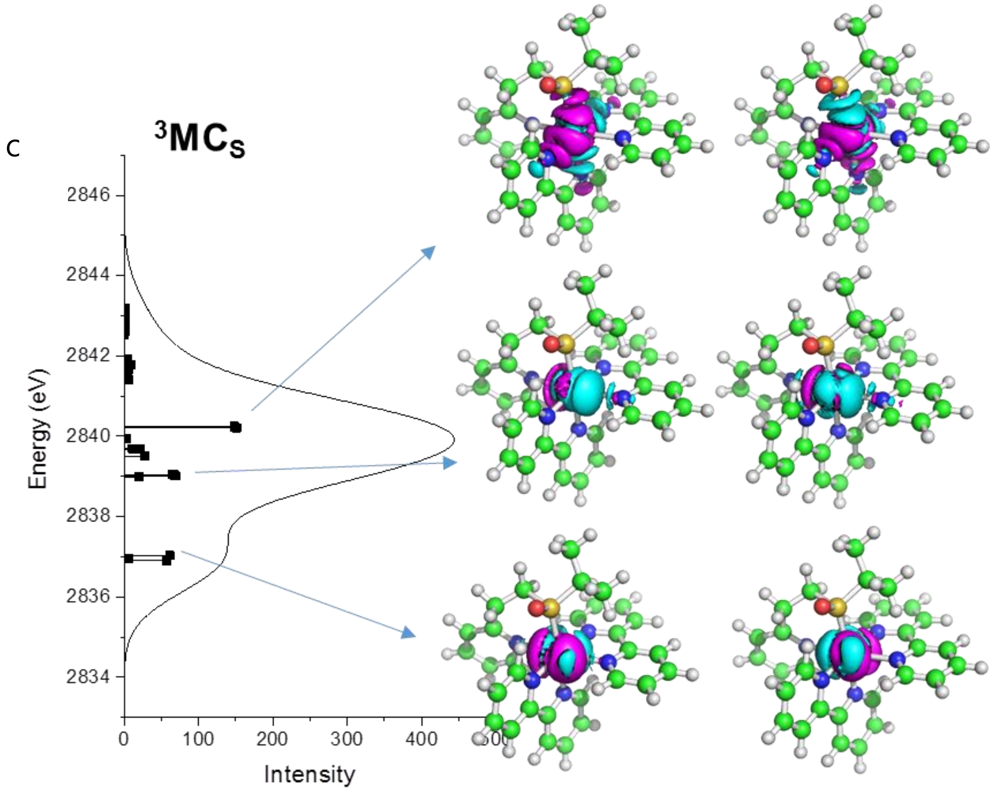
c
^3MC_S
Energy (eV)
2846
2844
2842
2840
2838
2836
2834
0
100
200
300
400
500
Intensity

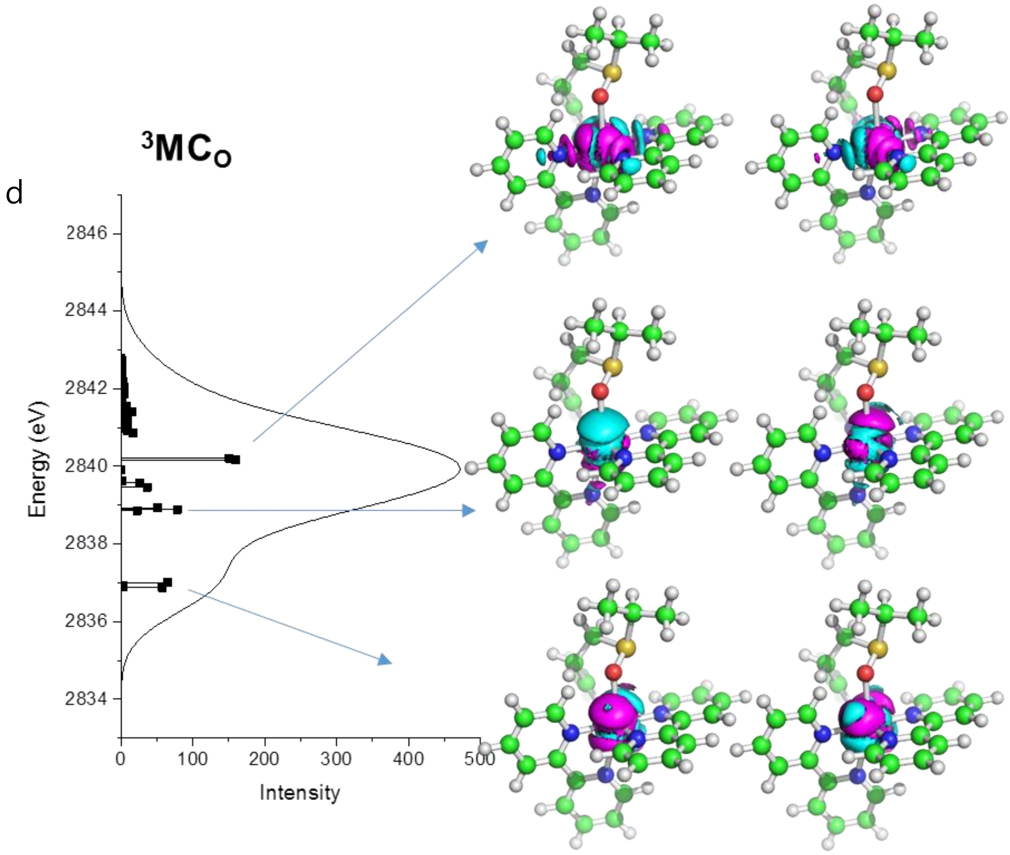
d
^3MC_O
Energy (eV)
2846
2844
2842
2840
2838
2836
2834
0
100
200
300
400
500
Intensity

e

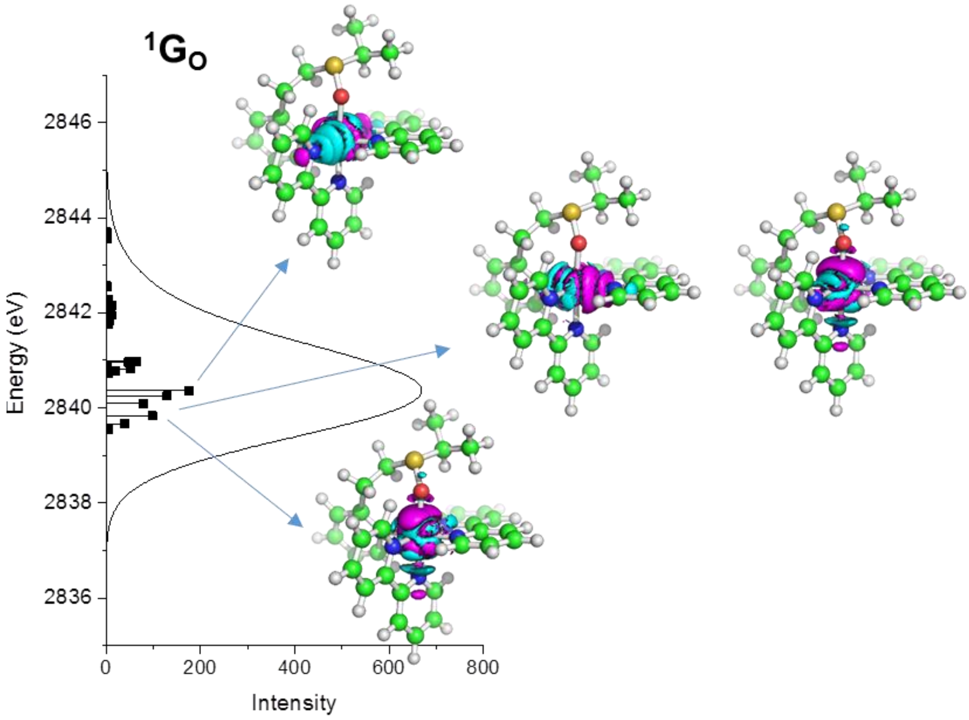

Supplementary Figure 2. TD-DFT simulated spectra at the S K-edge for all ground states and possible intermediate excited states, including the primary orbitals involved for the most intense transitions. (a) S-bonded ground state ^1G_S. (b) S-bonded ^{3}MLCT state $^3MLCT_S$. (c) S-bonded ^{3}MC state ^3MC_S. (d) O-bonded ^{3}MC state ^3MC_O. (e) O-bonded ground state ^1G_O.

a

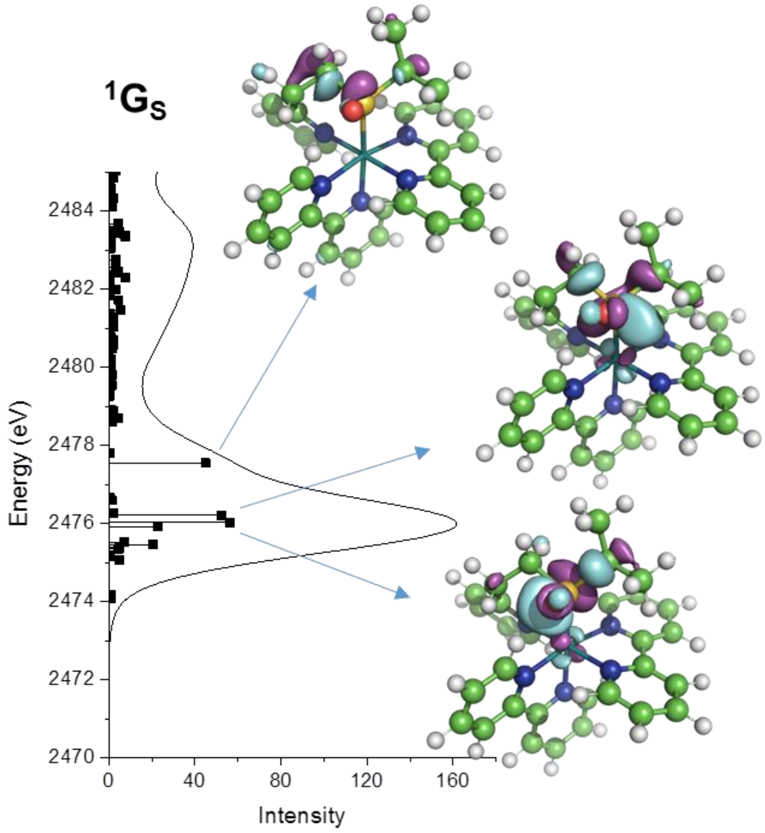

b

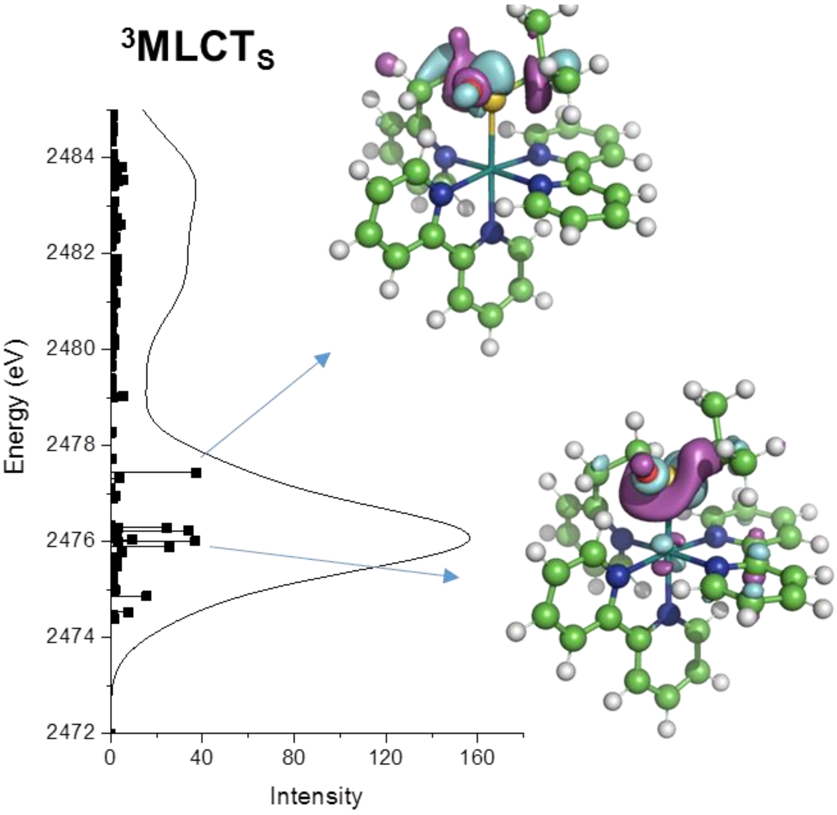

^{3}MLCT$_S$
Energy (eV)
2472
2474
2476
2478
2480
2482
2484
0
40
80
120
160
Intensity

c

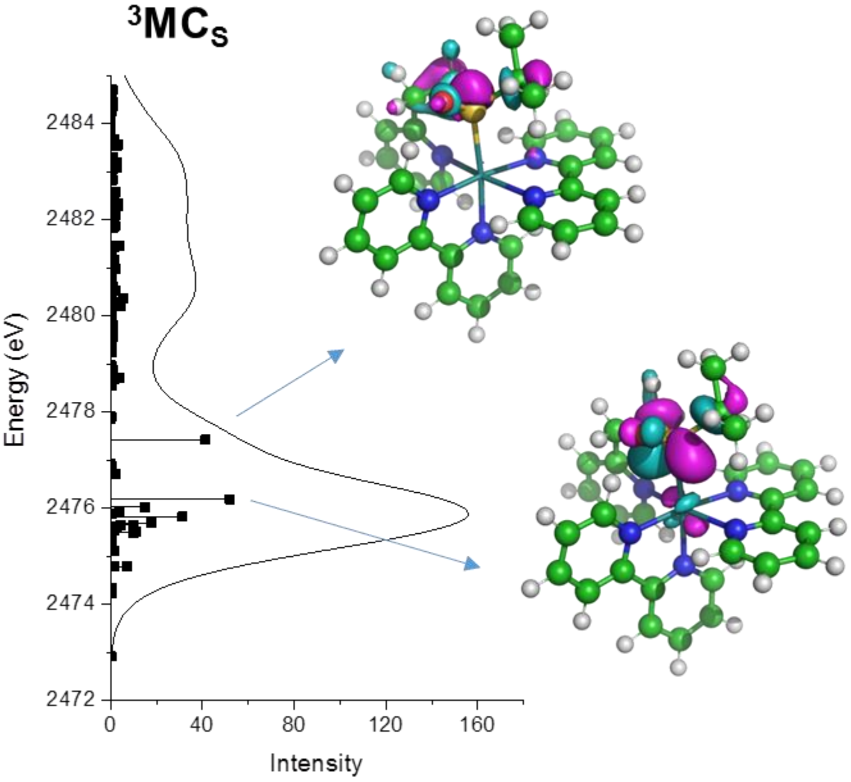

^{3}MC$_S$
Energy (eV)
2472
2474
2476
2478
2480
2482
2484
0
40
80
120
160
Intensity

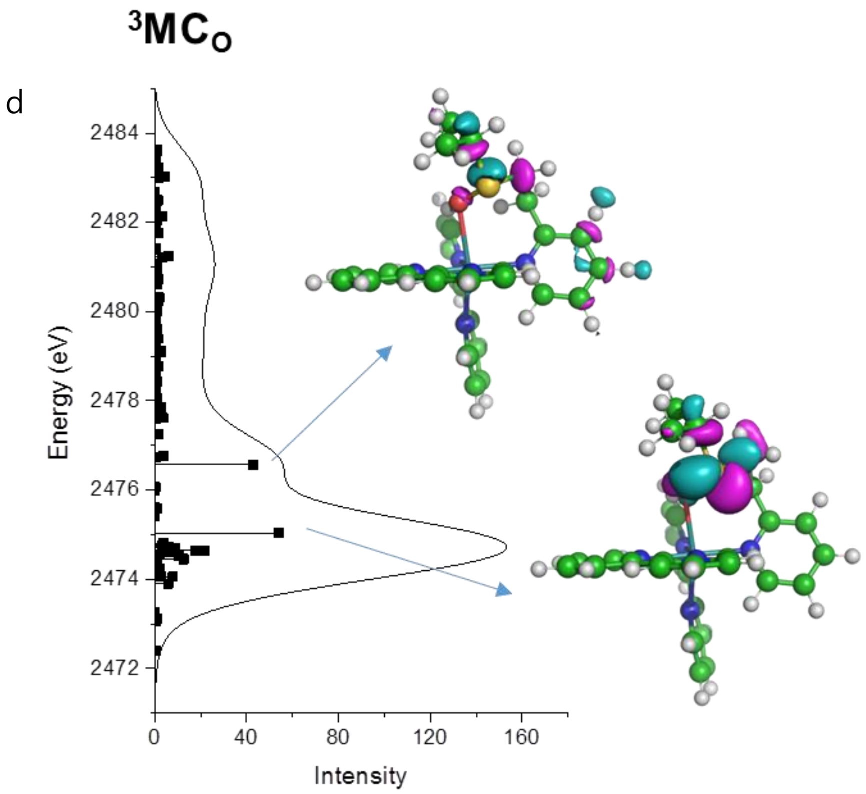

^3MC_O
d
Energy (eV)
2484
2482
2480
2478
2476
2474
2472
0
40
80
120
160
Intensity

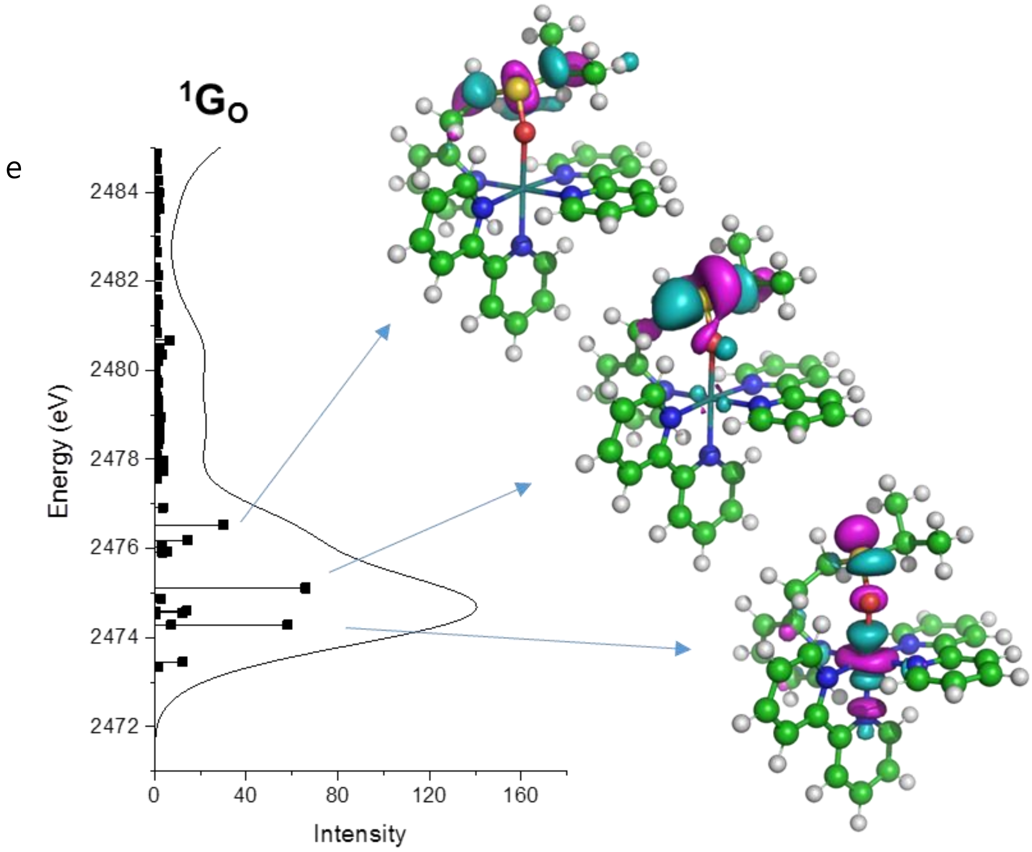

^1G_O
e
Energy (eV)
2484
2482
2480
2478
2476
2474
2472
0
40
80
120
160
Intensity

Supplementary Figure 3. Simulated S K-edge difference spectra. Spectra are overlaid to highlight the large differences in signal amplitudes (ΔA_{max}) expected between S- and O-bonded species and the comparable (large) signals expected for both ^3MC_O and ^1G_O states.

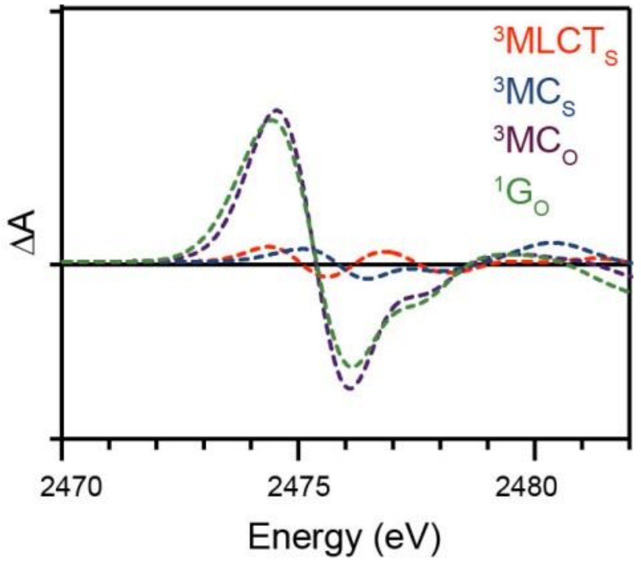

Supplementary Figure 4. Potential energy surface (PES) calculation results (relative to ^1G_S energy). The minimum potential energy for each possible intermediate excited state is indicated by the state name ($^3MLCT_{S/O}$, $^3MC_{S/O}$). Transition state energies are labeled 'TS' with dashed lines indicating transition pathways. Two minimum energy crossing points ($MECP_S$ and $MECP_O$, red dashed lines) were identified: 1) between the ^3MC_S and ^1G_S surfaces (5.0 kcal/mol barrier) 2) between the ^3MC_O and ^1G_O surfaces (3.5 kcal/mol barrier).

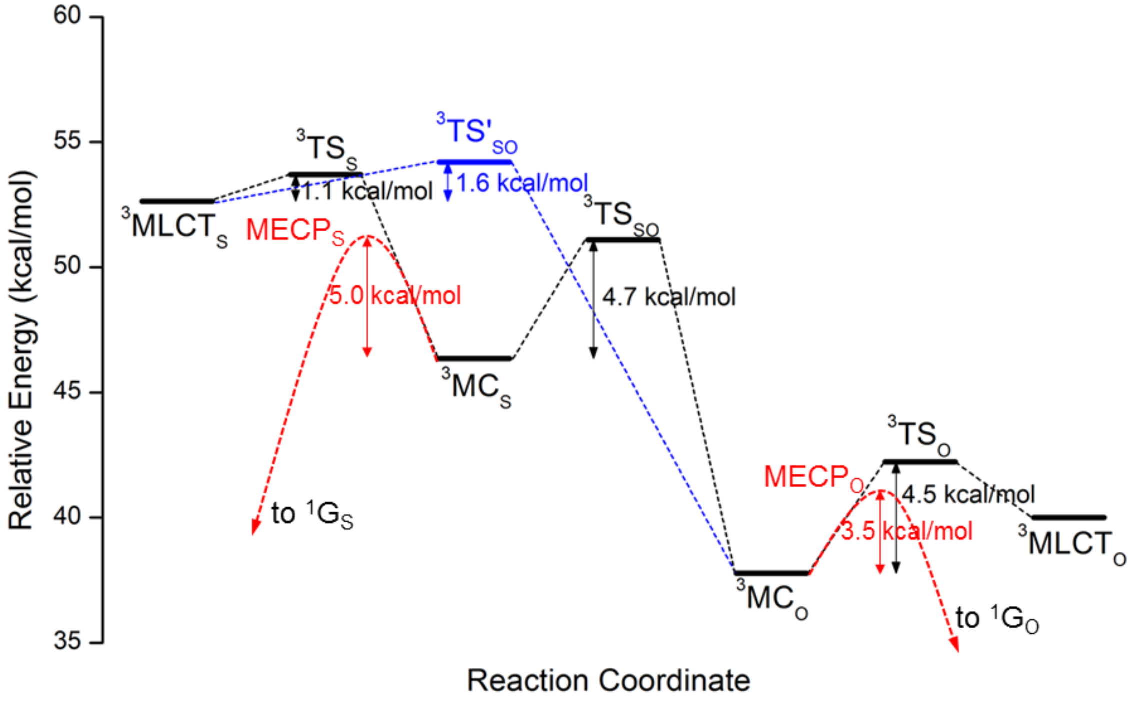

Supplementary Figure 5. Singular Value Decomposition (SVD) analysis of differential spectra. (a-b) SVD results for Ru L_3-edge spectra. One major and one minor spectral component are determined based on the singular values. **(c-d)** SVD results for S K-edge spectra. A single spectral contribution is determined.

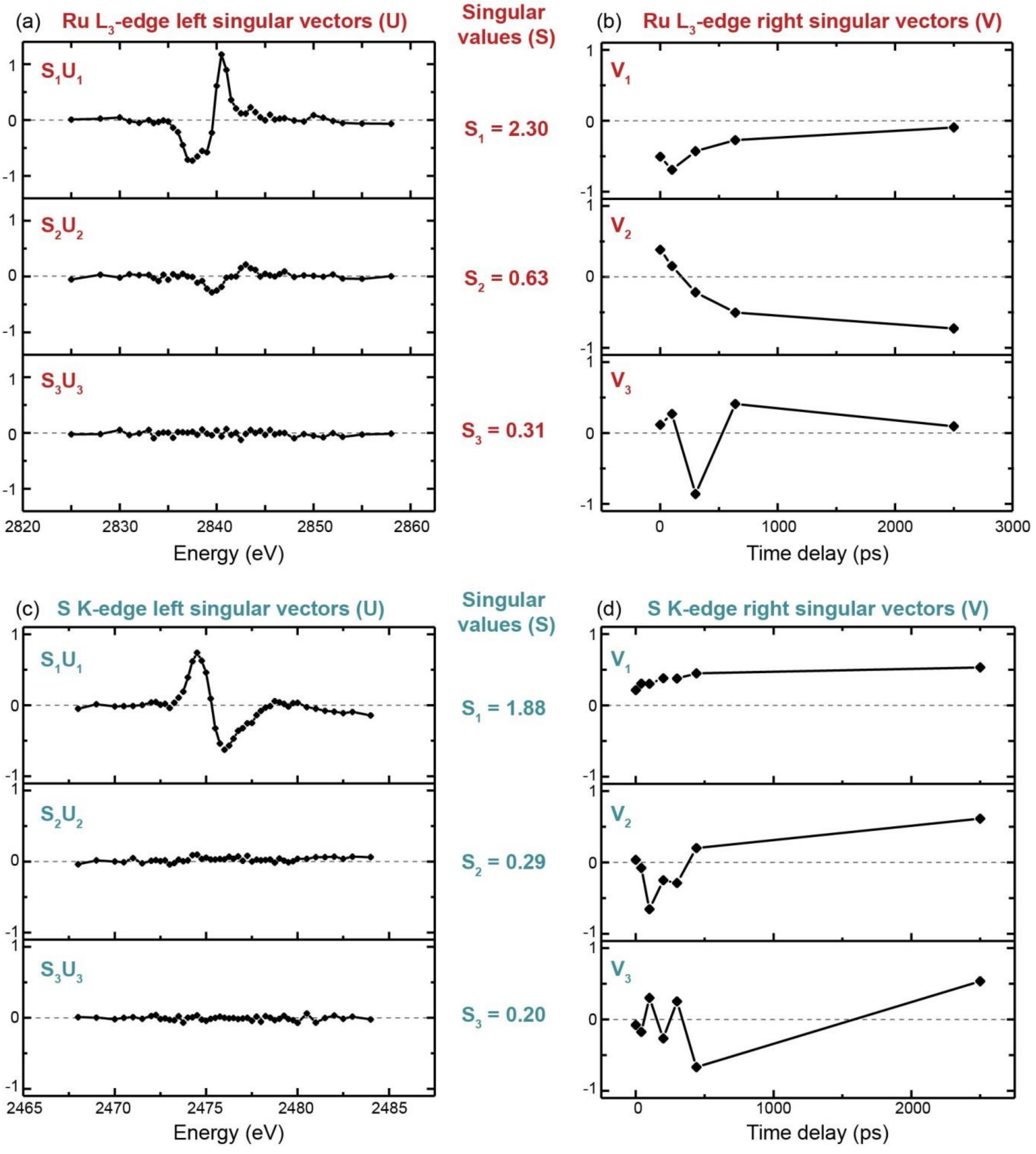

Supplementary Figure 6. Fractional population of each ground and excited state as a function of time.

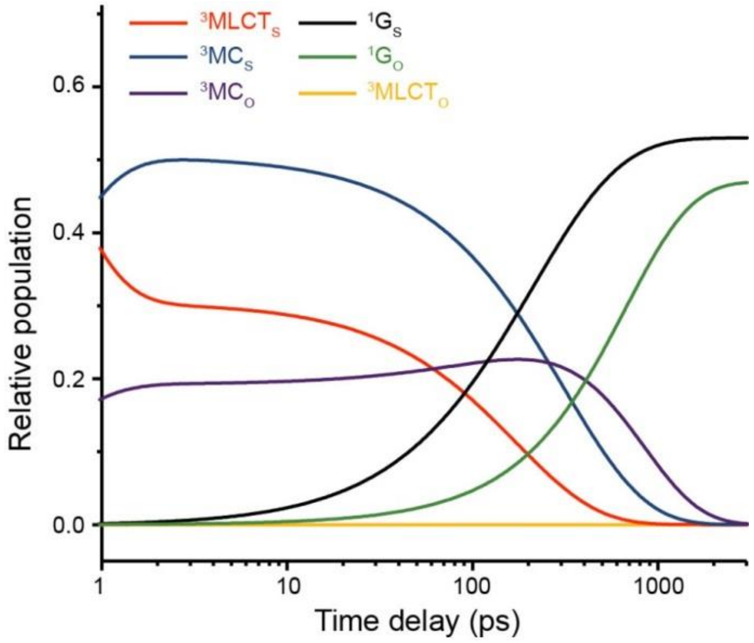

Supplementary Figure 7. Global data fit quality (chi-square) as a function of imposed formation times of the ^3MC_S and ^3MC_O states from $^3MLCT_S$

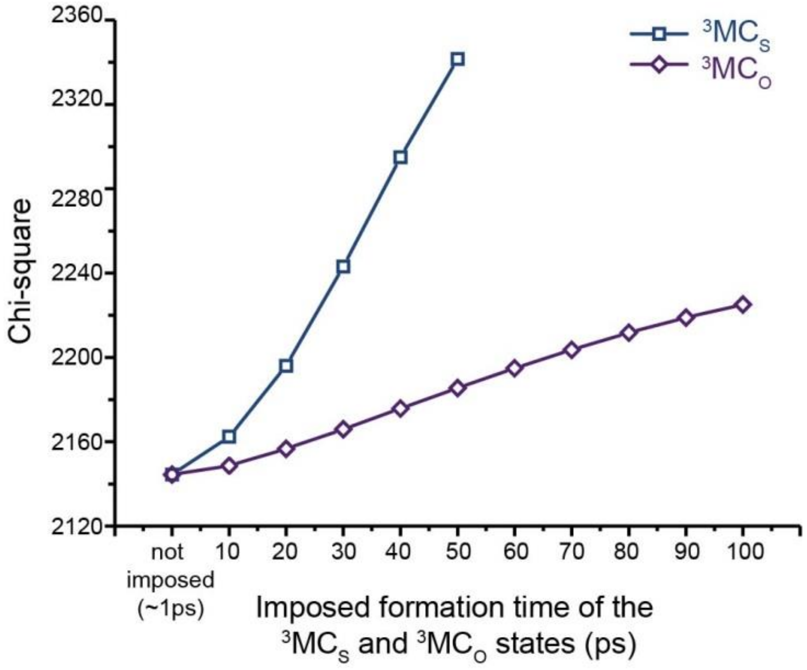

Supplementary Figure 8. Residuals of the Ru edge transient spectrum (100 ps delay) fit to a model that excludes the ^{3}MLCT$_{S'}$ intermediate and comparison to the TD-DFT simulated transient spectrum of ^{3}MLCT$_S$.

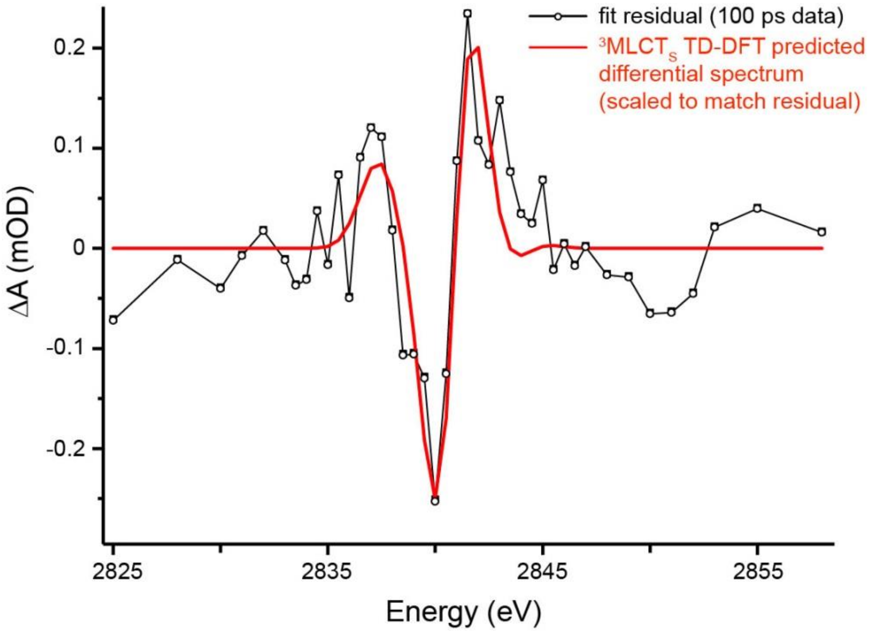

Supplementary Table 1. Selected bond lengths (Å) from the ruthenium center for the DFT optimized structures of all possible ground and transient intermediate states

	S	O	N(pyESO)	N_1(bpy$_1$)	N_2(bpy$_1$)	N_3(bpy$_2$)	N_4(bpy$_2$)
^{1}G$_S$	2.308	3.231	2.168	2.103	2.056	2.070	2.116
^{3}MLCT$_S$	2.372	3.249	2.199	2.091	2.026	2.036	2.117
^{3}MC$_S$	2.347	3.302	2.629	2.104	2.353	2.166	2.104
^{3}MC$_O$	3.382	2.508	2.170	2.403	2.069	2.081	2.143
^{3}MLCT$_O$	3.401	2.065	2.231	2.046	2.015	2.036	2.094
^{1}G$_O$	3.475	2.145	2.205	2.030	2.041	2.064	2.064

Supplementary Table 2. Potential energy minima calculation with solvent continuum. Minimum potential energy for each intermediate state calculated using the polarizable continuum model to account for the propylene carbonate solvent. No effect on the relative energies of the intermediate states was observed and the solvent was not considered further.

	Energy (kcal/mol), relative to ^{1}G$_S$		
	^{3}MLCT$_S$	^{3}MC$_S$	^{3}MC$_O$
**Gas phase**	52.63	46.35	37.77
**Propylene carbonate**	50.16	45.82	40.67

Supplementary Note 1. Global fitting procedure

To simulate the fractional population change of all intermediates, we set the following system of rate equations (Equations 1-8) and solved them (the $^3MLCT_{S'}$ state refers to a long-lived MLCT state observed in the Ru L_3-edge data, which is discussed in the main text and in Supplementary Figure 6). The entire experimental dataset (including all transient differential spectra and fixed-energy time-scans at the Ru and S edges) were globally fit to the kinetic model (Equations 1-8). The difference between the experimental data set and a theoretical dataset constructed from Equations 1-8 and the TD-DFT simulated spectra of each intermediate was minimized to obtain all rate constants.

$$\frac{\partial[^1G_S^*]}{\partial t} = -k_{^1G_S^* \to ^1MLCT_S}[^1G_S^*] \quad (1)$$

$$\frac{\partial[^1MLCT_S]}{\partial t} = k_{^1G_S^* \to ^1MLCT_S}[^1G_S^*] - k_{^1MLCT_S \to ^3MLCT_S}[^1MLCT_S] \quad (2)$$

$$\frac{\partial[^3MLCT_S]}{\partial t} = k_{^1MLCT_S \to ^3MLCT_S}[^1MLCT_S] - k_{^3MLCT_S \to ^3MC_S}[^3MLCT_S] - k_{^3MLCT_S \to ^3MC_O}[^3MLCT_S] - k_{^3MLCT_S \to ^3MLCT_{S'}}[^3MLCT_S] \quad (3)$$

$$\frac{\partial[^3MLCT_{S'}]}{\partial t} = k_{^3MLCT_S \to ^3MLCT_{S'}}[^3MLCT_S] - k_{^3MLCT_{S'} \to ^1G_S}[^3MLCT_{S'}] \quad (4)$$

$$\frac{\partial[^3MC_S]}{\partial t} = k_{^3MLCT_S \to ^3MC_S}[^3MLCT_S] - k_{^3MC_S \to ^3MC_O}[^3MC_S] - k_{^3MC_S \to ^1G_S}[^3MC_S] \quad (5)$$

$$\frac{\partial[^3MC_O]}{\partial t} = k_{^3MLCT_S \to ^3MC_O}[^3MLCT_S] + k_{^3MC_S \to ^3MC_O}[^3MC_S] - k_{^3MC_O \to ^1G_O}[^3MC_O] \quad (6)$$

$$\frac{\partial[^1G_S]}{\partial t} = k_{^3MLCT_{S'} \to ^1G_S}[^3MLCT_{S'}] + k_{^3MC_S \to ^1G_S}[^3MC_S] \quad (7)$$

$$\frac{\partial[^1G_O]}{\partial t} = k_{^3MC_O \to ^1G_O}[^3MC_O] \quad (8)$$

This least-squares fit was performed using the minimization package FMINUIT written by G. Allodi based on MINUIT written at CERN. The MINUIT routine minimizes the chi-square value defined as follows:

$$\chi^2 = \sum_j \sum_i \left(\frac{\langle \Delta A(E_j, t_i) \rangle - \Delta A_{exp}(E_j, t_i)}{\sigma_{i,j}} \right)^2 \quad (9)$$

where $\sigma_{i,j}$ is the measured experimental error. χ^2 was calculated from the entire experimental data set, including transient differential spectra and fixed energy time-scans for both Ru and S edges. Errors associated with each rate constant are also calculated by

MINUIT, representing one standard deviation. The fitting parameters included the rate constants from the rate equations above, the time delay corresponding to temporal overlap of X-ray and laser pulses, the X-ray temporal width, and scaling factors between the theoretical and experimental data (scaling of theory to experiment is done separately for Ru and S datasets). All four types of measurement (differential spectra and time-scans at Ru and S edges) were compared with theoretical curves, and the fit parameters were optimized.

Supplementary Note 2. Multiple photoisomerization mechanisms tested

Several possible photoisomerization mechanisms were tested to identify those models that best fit the experimental data. The model described in the main text (and corresponding to the kinetic equations listed above) resulted in the best fit and a chi-square value of 2144.47. The following alternative isomerization mechanisms were also tested:

1. Formation of an O-bonded MLCT state ($^3MLCT_O$) from the ^3MC_O state, followed by relaxation to the O-bonded ground state. The $^3MLCT_O$ state has been implicated in the possible adiabatic isomerization pathways of similar Ru-centered photochromic complexes.[1, 2] Upon inclusion of this additional pathway, the global data fit was largely unchanged (chi-square = 2144.50). Supplementary Figure 6 shows that no significant contribution of $^3MLCT_O$ was predicted at any time delay. Therefore, the $^3MLCT_O$ state was determined to be insignificant in the photoisomerization mechanism.

   MECP calculations comparing ^3MC_O and the O-bonded ground-state yield a transition energy of 3.5 kcal/mol for a non-adiabatic transition. The larger transition state energy of the adiabatic ^3MC_O to $^3MLCT_O$ pathway (4.5 kcal/mol) explains why no population of a $^3MLCT_O$ intermediate was observed from a theoretical perspective.

2. Formation of the $^3MLCT_S$ state was bypassed, resulting in a direct transition from $^1MLCT_S$ to ^3MC_S. This model resulted in a significantly worse fit quality (chi-square = 2634.55), largely affecting the Ru L_3-edge energy scans at short time delays.

3. Bifurcation was removed, such that only a single path to ^3MC_O formation was possible: Formation of the ^3MC_S state was bypassed (resulting in a direct transition from the $^3MLCT_S$ to the ^3MC_O state), which resulted in a significantly worse fit quality (chi-square = 9148.53). The direct formation of ^3MC_O from $^3MLCT_S$ was removed, such that

^3MC_O is only formed via the ^3MC_S state ($^3MLCT_S \rightarrow {}^3MC_S \rightarrow {}^3MC_O \rightarrow {}^1G_O$), which resulted in a significantly worse fit quality (chi-square = 2701.42).

Supplementary Note 3. Upper limit of MC state formation time

We note that the development of the S- and O-bonded MC states on the few picosecond time scale is considerably shorter than our experimental resolution. While the formation times of the MC states (indicated in Figure 5a) represent the best global fit, we also establish an upper limit by controlling the time constants for MC state growth and monitoring the global fit quality. The region of data most sensitive to the modeling of the fast isomerization dynamics is the rising portion of the Ru L-edge time scans at fixed energy. Supplementary Figure 7 illustrates how the fit quality becomes considerably worse as the simulated formation time of the MC states is increased beyond 10 ps.

Supplementary Note 4. Inclusion of $^3MLCT_{S'}$ state

In addition, a separate decay pathway from the $^3MLCT_S$ state back to the ^1G_S ground state, through a $^3MLCT_{S'}$ intermediate state, is included in the model and corresponding rate equations (Equations 1-8). Excluding this pathway from the model and fitting the Ru edge transient spectra resulted in residuals with clear ^3MLCT character. A visual comparison of these residuals (for the transient spectrum measured at 100 ps time delay) with the TD-DFT simulated differential spectra of the $^3MLCT_S$ state (calculated as $^3MLCT_S - {}^1G_S$) in Supplementary Figure 8 makes it clear that a long-lived ^3MLCT intermediate is required to adequately fit the data.

References

1. Göttle AJ, Dixon IM, Alary F, Heully J-L, Boggio-Pasqua M. Adiabatic Versus Nonadiabatic Photoisomerization in Photochromic Ruthenium Sulfoxide Complexes: A Mechanistic Picture from Density Functional Theory Calculations. *J Am Chem Soc* **133**, 9172-9174 (2011).

2. Vieuxmaire OPJ, Piau RE, Alary F, Heully J-L, Sutra P, Igau A*, et al.* Theoretical Investigation of Phosphinidene Oxide Polypyridine Ruthenium(II) Complexes: Toward the Design of a New Class of Photochromic Compounds. *The Journal of Physical Chemistry A* **117**, 12821-12830 (2013).
